# Concentrations of essential and non-essential elements in eastern North Pacific killer whales (*Orcinus orca*)

**DOI:** 10.1371/journal.pone.0353196

**Published:** 2026-07-15

**Authors:** Catherine F. Lo, Joseph K. Gaydos, Robert Poppenga, Lynne Barre, Kathy Burek-Huntington, John Calambokidis, Paul Cottrell, Debbie Duffield, Jessica L. Huggins, Dyanna M. Lambourn, Jim Rice, Heindrich N. Snyman, Judy St. Leger, Matthew Van Daele, Kristi L. West, Forrest M. Gomez, Stephen Raverty

**Affiliations:** 1 The SeaDoc Society, Karen C. Drayer Wildlife Health Center - Orcas Island Office, UC Davis Weill School of Veterinary Medicine, Eastsound, Washington, United States of America; 2 California Animal Health and Food Safety Laboratory System, University of California Davis, Davis, California, United States of America; 3 West Coast Regional Office, National Marine Fisheries Service, National Oceanic and Atmospheric Administration (retired), Seattle, Washington, United States of America; 4 Alaska Veterinary Pathology Services, Eagle River, Alaska, United States of America; 5 Cascadia Research Collective, Olympia, Washington, United States of America; 6 Fisheries and Oceans Canada, Fisheries and Aquaculture Management, Vancouver, British Columbia, Canada; 7 Biology Department, Portland State University, Portland, Oregon, United States of America; 8 Washington Department of Fish and Wildlife, Marine Mammal Investigations, Lakewood, Washington, United States of America; 9 Marine Mammal Institute, Oregon State University, Newport, Oregon, United States of America; 10 Animal Health Laboratory, University of Guelph, Kemptville, Ontario, Canada; 11 Cornell University, Ithaca, New York, United States of America; 12 Natural Resources Department, Sun’aq Tribe of Kodiak, Kodiak, Alaska, United States of America; 13 Health and Stranding Lab, Human Food, Nutrition and Animal Sciences, University of Hawai’i at Mānoa, Honolulu, Hawai’i, United States of America; 14 National Marine Mammal Foundation, San Diego, California, United States of America; 15 Animal Health Centre, Ministry of Agriculture, Abbotsford, British Columbia, Canada; King Faisal Specialist Hospital and Research Center, SAUDI ARABIA

## Abstract

Essential and non-essential elements can harm marine wildlife and impact ecosystems. Using liver (n = 35) and kidney (n = 17) samples from 35 animals from three distinct killer whale (*Orcinus orca*) ecotypes (fish-eating residents, mammal-eating transients, and offshore shark-eating specialists) stranded from California to Alaska and Hawaii, we determined elemental concentrations (arsenic (As), barium (Ba), beryllium (Be), calcium (Ca), cadmium (Cd), cobalt (Co), copper (Cu), chromium (Cr), iron (Fe), mercury (Hg), methylmercury (MeHg), manganese (Mn), molybdenum (Mo), magnesium (Mg), nickel (Ni), lead (Pb), selenium (Se), thallium (Tl), vanadium (V), and zinc (Zn)). Also, we evaluated associations with demographic and genetic factors. Calves had lower Cd than other life stages in kidney and liver tissue. Adult females had higher liver Se than calves and higher total mercury (tHg) in kidney and liver than calves. Adult males had higher liver Cd, tHg, and Se than calves. Residents had higher liver Mg, Mn, and Mo than transients. Linear regression showed life stage had some effect on the concentration of kidney Hg, and on the concentration of liver Mg, Mn, Mo, Cd, Hg, and Se. A prior study found no microscopic evidence of toxicosis in the tissues of examined animals. Population had some effect on liver Cd and Mg. Mean ± SD Se:Hg molar ratios in kidney (0.871 ± 0.645) and liver (0.932 ± 0.940) were consistent with prior research (at or nearly 1:1), but ratios based on factors varied in kidney. These results expand knowledge of elements in northeastern Pacific killer whales.

## Introduction

Elements are complex and can be found naturally in the environment or sourced from anthropogenic activities. Essential elements (*e.g.,* calcium (Ca), cobalt (Co), copper (Cu), iron (Fe), manganese (Mn), magnesium (Mg), molybdenum (Mo), selenium (Se), zinc (Zn)) perform critical roles in animals (*e.g.,* energy metabolism, iron absorption, post-natal growth, protection against oxidation, enzymatic functions, homeostasis [1–3]) and can be toxic at high concentration [3–5]. In comparison, non-essential elements (*e.g.,* arsenic (As), cadmium (Cd), lead (Pb), mercury (Hg), thallium (Tl)) have no biological function and are toxic at low concentrations [3–7]. While “normal” reference ranges for some elemental concentrations have been identified in a few marine mammal species (*e.g.*, striped dolphins (*Stenella coeruleoalba*) [8]; white-beaked dolphins (*Lagenorhynchus albirostris*) [9,10]; bottlenose dolphins (*Tursiops truncatus*) [11]), there are some essential elements without established “normal” reference ranges.

The pathologic consequences of elemental toxicosis in marine mammals are unclear [12,13], but relationships have been identified between high concentrations of some elements and pathology. For example, bottlenose dolphins (*Tursiops aduncus*) in South Australia with renal pathology had significantly higher hepatic concentrations of Cu, Cd, and Zn [14]. Notably, two animals with high elemental burdens displayed a range of diseases, including renal pathology, bone malformations, and elevated metallothioneins which were pre-defined as markers of elemental toxicity [14,15]. Blood from captive belugas (*Delphinapterus leucas*) exposed to high concentrations of inorganic and organic Hg showed decreased immune function, including lower production of T lymphocytes [16,17]. Immunotoxicity has also been demonstrated in dose-response relationships between several elements (*e.g.,* total mercury (tHg), methylmercury (MeHg), Cd) and T-cell response in other marine mammals [17]. Common dolphins (*Delphinus delphis*) stranded off Portugal’s Iberian Peninsula with higher concentrations of tHg, Se, and Cd demonstrated more pathologic lesions, including bronchopneumonia, stomach ulceration, lung adhesions, and liver abscesses, as well as higher parasite burdens [12].

Concentrations of elements in odontocetes often differ by multiple dependent factors including species, distribution, sex, age, and prey preferences [5,6,12,13,18–24]. Because so many factors are correlated, separating the multiple potential covariates responsible for elemental accumulation and disease can be challenging, especially without well-designed case-control studies. Weak but interesting associations have been shown with elements for species, physiology, diet, and spatial distribution [12,25–29]. For example, accumulation of some nutritionally relevant elements (*e.g.,* Cu, Se) and contaminants (*e.g.,* tHg, Zn, vanadium (V)) in the liver of bottlenose dolphins from the southeastern United States was seen to correlate with age [20]. Age accumulation, however, can also be species dependent where As concentrations increased with age in Risso’s dolphins (*Grampus griseus*) and melon-headed whales (*Peponocephala electra*), but not in finless porpoise (*Neophocaena phocaenoides*), harbor porpoise (*Phocoena phocoena*), Indo-Pacific humpback dolphins (*Sousa chinensis*), killer whales (*Orcinus orca*), or short-finned pilot whales (*Globicephala macrorhynchus*) [18]. Sex can be a co-factor, but not always. For example, in the same study [18] sex-associated elevations in As were demonstrated for male Risso’s dolphins, but not in other species. Despite there being some associations between factors and elemental concentrations, occasionally no correlation has been detected, as was the case with gray whales (*Eschrichtius robustus*) from the United States Pacific Coast [30], and with Guiana dolphin (*Sotalia guianensis*), Atlantic spotted dolphin (*Stenella frontalis*), rough-toothed dolphin (*Steno bredanensis*), bottlenose dolphin, franciscana dolphin (*Pontoporia blainvillei*), pygmy killer whale (*Feresa attenuata*), and a killer whale from the coast of southeastern Brazil [6].

Relatively little is known about concentrations of elements or the role they play in killer whale health [5,6,18,31–33]. A few studies have measured some elements (*e.g.,* As, Cu, Cd, tHg, Se:Hg, Mn, Pb, Se, strontium (Sr)) in killer whales that stranded in the United Kingdom [31], Japan [18], Hawaii [5], Brazil [6], and British Columbia [34,35]. Endo et al. [32,33] examined liver, kidney, and muscle from nine seal and squid eating killer whales that stranded off the coast of northern Japan for tHg, MeHg, Se, Cd, Zn, Cu, Fe, and Mn concentrations. While postmortem examination found no significant pathologic lesions, they did show mature whales had higher tissue concentrations of tHg, MeHg, Se, Cd, and Fe than did calves, while calves had higher concentrations of Mn and Cu [32,33]. This could be related to differences in foraging or metabolism [12,23,25,36]. Also, it could be due to offloading of contaminants by mothers to calves, which is known to occur with persistent organic pollutants. Offloading of some elements has occurred in a few marine mammal species like Peruvian fur seals (*Arctocephalus australis*) [37] and West Indian manatees (*Trichechus manatus*) [38], but it is not known if offloading occurs in killer whales.

Killer whales are the most widely distributed marine mammal in the world, are top predators, and are long-lived with females living up to 80–90 years of age [39]. Additionally, different populations and ecotypes have varied diets. These factors make them ideal candidates to enhance our understanding of the pathways of element exposure. In the eastern North Pacific, killer whale populations are distinguished by ecotypes, with two recently proposed as distinct species [40]. Residents are primarily fish (salmon) eaters, transients (also called Bigg’s) primarily prey on marine mammals, and offshores consume fish, specializing in sharks [4,41]. In addition to diet, each ecotype has unique acoustics, genetics, and behaviors [41]. Exposure to elements likely varies across ecotypes, by location, and with age. Furthermore, elucidating elemental concentrations is critical for determining if they play a clinical or subclinical role in the health of endangered Southern Resident killer whales (SRKWs) [42]. Using liver and kidney samples collected from stranded killer whales [43], we analyzed concentrations of twenty elements (As, barium (Ba), beryllium (Be), Ca, Cd, Co, Cu, chromium (Cr), Fe, Hg, MeHg, Mn, Mo, Mg, nickel (Ni), Pb, Se, Tl, V, Zn) and looked for demographic, spatial, ecotype, and population level associations in concentrations as well as investigated the relationship between Se and Hg concentrations in killer whales in the northeastern Pacific Ocean from California to Alaska and Hawaii.

## Materials and Methods

### Sample collection

**Fig 1:**
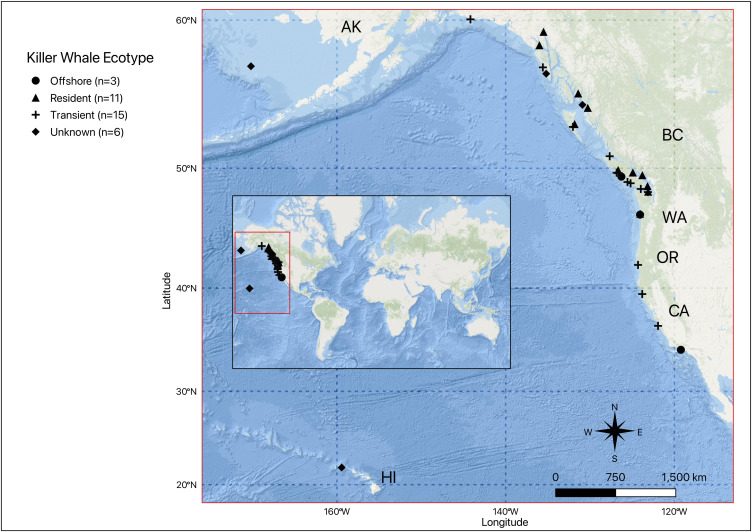
Stranding locations of the 35 killer whales by ecotype in the United States West Coast Alaska, Hawaii, and British Columbia. Offshore ecotype is denoted by circles (n = 3). Resident ecotype is denoted by triangles (n = 11). Transient ecotype is denoted by plus signs (n = 15). Unknown ecotype is denoted by diamonds (n = 6). Map created using the Free and Open Source QGIS Geographic Information System under a GNU General Public License, original copyright 1991.

As part of a larger effort to understand diseases of killer whales in the Eastern North Pacific [43,44], intensive efforts were made to examine 35 dead killer whales from 1997–2022 that stranded from California to Alaska and Hawaii [43] (Fig 1). Liver and kidney samples collected from these animals were primarily stored in tinfoil with fewer stored in whirl packs, frozen at −80°C, and then tested for essential and non-essential elements. Kidney was sampled in 17 of 35 animals and liver in all animals. Elemental concentrations were determined and separated based on demographics (Table 1) including by sex (female, male, unknown), ecotype (Transient, Resident, Offshore [43,45]) and when possible, by population (Alaska Resident; AR, Southern Resident; SR, Northern Resident; NR, Offshore; O, West Coast Transient; WCT, Gulf of Alaska Transient; GAT [46]) determined through photo-identification, stomach contents, or genetic sequencing [43,47]. Home ranges vary across populations [43,45]. The AR killer whales can be found from southeastern Alaska to the Bearing Sea’s Aleutian Islands. The SR and NR killer whales are found in coastal and inland waters from southeastern Alaska to Monterey Bay, California, and the outer coast of Washington State. Transient killer whale populations vary within the subpopulations, but they range within the Bering Sea to California. The offshore killer whale population focuses on the continental shelf from the Aleutian Islands to California [43,45]. Life stage, or what killer whale biologists termed stage class, was assigned with body length as a proxy for age [48] with slight modification. Due to limited samples (data), some categories were lumped or combined: calves (0–1 years old; n = 17), juveniles (1–9 years old; n = 8), adult females (ages 10 + ; n = 4), adult males (ages 10 + , n = 5), and adult with unknown sex (ages 10 + ; n = 1). Elemental concentrations were evaluated by sex, life stage, and population, as well as by cause of mortality [43].

**Table 1:** Demographic data for kidney and liver sampled from 35 stranded killer whales.

		Kidney	Liver
Sex	Female	9	16
	Male	7	16
	Unknown	1	3
Life Stage	Adult Female	2	4
	Adult Male	3	5
	Adult Unknown	0	1
	Juvenile	5	8
	Calf	7	17
Ecotype	Offshore	0	3
	Resident	8	12
	Transient	4	15
	Unknown	5	5
Population	Alaska Resident (AR)	0	1
	Southern Resident (SR)	4	6
	Northern Resident (NR)	3	4
	Resident (Unspecified)	1	1
	Offshore (O)	0	3
	Gulf of Alaska Transient (GAT)	0	2
	West Coast Transient (WCT)	2	8
	Transient NT1 haplotype (T NT1)	1	1
	Transient (Unspecified)	1	4
	Unknown	5	5
	Total (N)	17	35

### Elemental analyses

Analysis was performed at one of four laboratories: California Animal Health & Food Safety Lab System (CAHFS) in Davis, CA, United States, Animal Health Centre (AHC) in Abbottsford, British Columbia, Canada, Prairie Diagnostic Services (PDS) in Saskatoon, Saskatchewan, Canada, or ALS Environmental (previously known as Columbia Analytical Services) in Kelso, WA, United States. Tissue analysis at CAHFS was completed by digesting samples with 3 ml of nitric acid followed by a mixture of 2 ml of hydrochloric acid and 18MoHm water to bring to a final volume of 10mL. Elements (As, Ba, Be, Cd, Co, Cu, Cr, Fe, Hg, Mn, Mo, Ni, Pb, Tl, V, Zn) were measured in prepared tissue samples using inductively coupled plasma optical emission spectroscopy (ICP-OES). Selenium concentrations were determined by diluting digested tissue with a separate mixture and were measured using inductively coupled plasma mass spectrometry (ICP-MS). Methylmercury concentrations were quantified by extracting a final solution from homogenized samples that were placed in a water bath, shaken, and filtered, and were measured using high performance liquid chromatography-inductively coupled plasma mass spectrometry (HPLC-ICP-MS). Moisture (%) was measured by drying samples at 95°C for a minimum of 12 hours then cooled. This was calculated by dividing the difference between sample wet weight and sample dry weight by the wet weight value. All elements, excluding MeHg and percent moisture, were reported in micrograms per gram (µg/g) wet weight (ww). The National Research Council Canada quality control (QC) and assurance criteria were used as the basis for all analyses. Tissue analysis and QC performed at AHC [7], ALS Environmental [49–51], CAHFS [52–54], and PDS [55,56] followed protocols outlined to their respective laboratories. Results from AHC were reported in ug/g dry weight (dw), and ALS Environmental and PDS were reported in ug/g ww. Prolonged storage of samples in subzero freezers could have led to varying moisture content. To standardize the data, ww results were converted to dw. Reporting limits were not adjusted due to different reporting methods. A mean moisture content of 70% was assumed for samples when the moisture content was not analyzed [57].

### Statistical analyses

Samples with concentrations below detection limits (BDLs) were assigned one-half the respective detection limit. Summary statistics (*e.g.,* raw mean, median, standard deviation, range) were calculated for each element. Kidney and liver Se:Hg molar ratios were determined by dividing Se and Hg concentrations by their respective atomic weight (Se = 78.96, Hg = 200.59) and then by dividing µmol concentration of Se by the µmol concentration of Hg. The percent of MeHg from tHg for both tissues as well as by life stage was determined by dividing the concentration of MeHg by the concentration of tHg times 100. Methylmercury was converted from ug/L to ug/g dw to align all units for this analysis. Finally, linear regression was used to investigate the relationship between log transformed Se and Hg as well as Se and MeHg ug/g dw concentrations to meet assumptions of normality. Kidney Ba, Be, Cr, Co, Pb, Mo, Ni, and Tl, and liver Ba, Be, Cr, Co, Pb, Ni, and Tl were removed from analyses because all concentrations were BDLs, or too few concentrations were above the detection limits (< 3 reported values).

Elements were tested for normality and homoscedasticity using the Shapiro-Wilk’s test and Bartlett’s test, respectively. Pairwise comparisons using the Kruskal-Wallis rank sum test were used to identify significant differences between contaminant concentrations and each covariate. Effect size measure (ε^2^) for each pairwise comparison was calculated to show the strength of association. A post hoc Dunn’s test with Holm correction was used to determine which groups significantly differ. Log transformations were performed to meet assumptions of normality for elements (Kidney: Cd and Hg; Liver: Cd, Cu, Hg, Mg, Mn, Mo, and Se) with non-gaussian distributions. A generalized linear model (GLM) was used to investigate the effect of explanatory variables on contaminant concentrations in kidney and liver. Model selection was determined using the lowest Akaike Information Criteria (AIC) value (score of delta < 2) [58] and validation involved checking assumptions of normality and model residuals [59]. Statistical analyses were performed using R v4.2.1 [60] and used a p-value < 0.05 for statistical significance (ɑ < 0.05).

## Results

**Fig 2:**
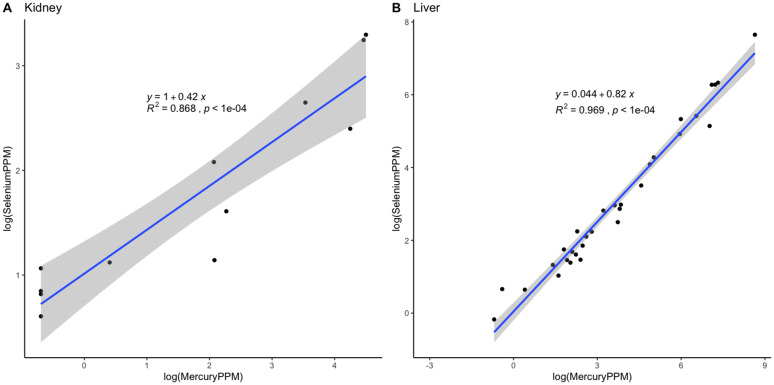
Simple linear regression and 95% confidence interval bands comparing log transformed Se and Hg showed a significant linear correlation in (A) kidney and (B) liver.

Concentrations of elements in kidney and liver are reported in parts per million (ppm) dw except for MeHg (where results are reported in parts per billion dw) (Tables 2 and 3). Simple linear regression between log transformed Se and Hg showed a strong positive correlation in both kidney (R^2^ = 0.868, p < 0.0001; Fig 2A) and liver (R^2^ = 0.969, p < 0.0001; Fig 2B). For animals with both Se and Hg data in kidney (n = 12) and liver (n = 31), the Se:Hg molar ratio mean ± standard deviation and range in parenthesis was 0.871 ± 0.645 (0.399–14.7) and 0.932 ± 0.940 (0.388–4.27), respectively. Variability in molar ratios based on demographic variables for kidney and liver are shown in Tables 4 and 5. Simple linear regression between log transformed Se and MeHg showed a positive correlation in the liver (R^2^ = 0.292, p = 0.011), but not in kidney (R^2^ = 0.165, p = 0.497). Median %MeHg in kidney and liver was 13.1% (n = 6) and 8.16% (n = 22), respectively. Summary data (median ± standard deviation, sample size) of %MeHg based on life stage are reported in ppm dw (Table 6).

**Table 2:** Concentrations of elements determined for kidney tissue. Values are reported in ppm dw unless otherwise noted.

	n	Mean ± SD	Median	Min	Max
**Arsenic**	15	0.859 ± 1.43	0.500	0.203	6.00
**Barium**	4	0.0804 ± 0.111	0.0250	0.0250	0.247
**Beryllium**	4	0.0250 ± 0	0.0250	0.0250	0.0250
**Cadmium**	15	18.9 ± 54.5	0.700	0.0250	213
**Calcium**	5	1277 ± 1722	485	247	4333
**Cobalt**	4	0.0813 ± 0.113	0.0250	0.0250	0.250
**Copper**	16	11.8 ± 6.11	10.8	3.80	25.3
**Chromium**	4	0.330 ± 0.624	0.0250	0.0050	1.27
**Iron**	14	454 ± 201	433	130	800
**Lead**	15	0.343 ± 0.231	0.500	0.0250	0.500
**Mercury**	17	24.2 ± 31.4	8.00	0.500	90.0
Methylmercury*	6	4262 ± 7141	983	1.98	18333
**Magnesium**	5	448 ± 271	367	89.0	767
**Manganese**	15	1.97 ± 1.10	1.63	0.700	5.33
**Molybdenum**	14	0.151 ± 0.0804	0.200	0.0250	0.200
**Nickel**	5	0.371 ± 0.595	0.0250	0.0250	1.40
**Selenium**	12	8.86 ± 9.03	4.07	1.83	27.0
**Thallium**	4	0.0438 ± 0.0125	0.0500	0.0250	0.0500
**Vanadium**	4	0.295 ± 0.226	0.187	0.173	0.633
**Zinc**	16	95.5 ± 50.2	86.7	34.0	203

*parts per billion (ppb) dw

**Table 3:** Concentrations of elements determined for liver tissue. Values are reported in ppm dw unless otherwise noted.

	n	Mean ± SD	Median	Min	Max
**Arsenic**	28	0.582 ± 0.346	0.500	0.500	2.33
**Barium**	17	0.0482 ± 0.0100	0.0500	0.0250	0.0700
**Beryllium**	17	0.0120 ± 0.0050	0.0100	0.0100	0.0250
**Cadmium**	33	6.28 ± 30.7	0.150	0.100	177
**Calcium**	12	296 ± 474	103	6.67	1727
**Cobalt**	18	0.133 ± 0.0513	0.150	0.0250	0.215
**Copper**	34	78.0 ± 85.2	48.7	3.00	332
**Chromium**	17	0.280 ± 0.433	0.150	0.0050	1.86
**Iron**	33	729 ± 423	712	128	1643
**Lead**	33	0.562 ± 0.243	0.500	0.0250	1.00
**Mercury**	35	374 ± 1014	16.4	0.0500	5667
Methylmercury*	22	5062 ± 7152	1573	1.83	26733
**Magnesium**	12	505 ± 592	323	1.85	2220
**Manganese**	34	6.18 ± 5.20	4.87	0.612	28.7
**Molybdenum**	27	0.509 ± 0.727	0.200	0.200	3.10
**Nickel**	18	0.290 ± 0.0812	0.300	0.0250	0.430
**Selenium**	31	155 ± 396	12.2	0.840	2100
**Thallium**	17	0.457 ± 0.165	0.500	0.0250	0.700
**Vanadium**	17	0.581 ± 0.579	0.215	0.150	1.67
**Zinc**	34	276 ± 245	183	1.70	1000

*parts per billion (ppb) dw

**Table 4:** Se:Hg molar ratios (mean, standard deviation, range, and sample size) in kidney tissue based on demographic variables.

		Se:Hg ratio	Standard deviation	Range	Sample size
Sex	Female	6.84	6.42	0.762–14.7	4
	Male	3.98	5.27	0.399–11.9	7
	Unknown Sex	5.19	–	5.19	1
Life Stage	Adult Female	0.762	–	0.762	1
	Adult Male	0.814	0.361	0.399–1.05	3
	Adult Unknown	–	–	–	–
	Juvenile	1.54	0.926	0.752–2.56	3
	Calf	10.5	3.55	5.19–14.7	5
Ecotype	Offshore	–	–	–	–
	Resident	3.30	4.11	0.399–11.9	7
	Transient	7.29	5.52	1.05–11.5	3
	Unknown Ecotype	7.74	9.89	0.752–14.7	2
Population	AR	–	–	–	–
	SR	2.29	2.14	0.399–5.19	4
	NR	6.31	7.84	0.762–11.9	2
	Resident (Unspecified)	1.31	–	1.31	1
	O	–	–	–	–
	GAT	–	–	–	–
	WCT	10.4	1.56	9.31–11.5	2
	T NT1	1.05	–	1.05	1
	Transient (Unspecified)	–	–	–	–
	Unknown Population	7.74	9.89	0.752–14.7	2

**Table 5:** Se:Hg molar ratios (mean, standard deviation, range, and sample size) in liver tissue based on demographic variables.

		Se:Hg ratio	Standard deviation	Range	Sample size
Sex	Female	1.60	1.02	0.388–4.27	14
	Male	1.29	0.454	0.739–2.40	14
	Unknown Sex	3.60	3.34	0.991–7.37	3
Life Stage	Adult Female	0.980	0.446	0.388–1.45	4
	Adult Male	1.07	0.204	0.823–1.31	5
	Adult Unknown	0.991	–	0.991	1
	Juvenile	2.35	1.71	0.739–1.70	7
	Calf	1.14	0.333	0.995–7.37	14
Ecotype	Offshore	2.23	1.04	1.15–3.22	3
	Resident	2.01	1.85	0.876–7.37	11
	Transient	1.33	0.993	0.388–4.27	12
	Unknown Ecotype	1.28	0.195	0.939–1.42	5
Population	AR	0.876	–	0.876	1
	SR	2.66	2.38	1.12–7.37	6
	NR	1.41	0.408	0.941–1.68	3
	Resident (Unspecified)	1.07	–	1.07	1
	O	2.23	1.04	1.15–3.22	3
	GAT	0.991	–	0.991	1
	WCT	1.70	1.29	0.739–4.27	6
	T NT1	0.907	–	0.907	1
	Transient (Unspecified)	0.976	0.545	0.388–1.70	4
	Unknown Population	1.28	0.195	0.939–1.42	5

**Table 6:** Proportion of MeHg (median %MeHg, standard deviation, sample size) in kidney and liver based on life stage.

Life Stage	Tissue	Median % MeHg (ppm dw)	Standard deviation	Sample size
Adult Female	kidney	5.56	–	1
	liver	10.6	14.6	2
Adult Male	kidney	20.4	–	1
	liver	1.00	0.308	3
Adult Unknown	kidney	–	–	–
	liver	1.54	–	1
Juvenile	kidney	5.79	10.9	3
	liver	2.82	8.72	5
Calf	kidney	22.2	–	1
	liver	15.2	30.4	11

**Fig 3:**
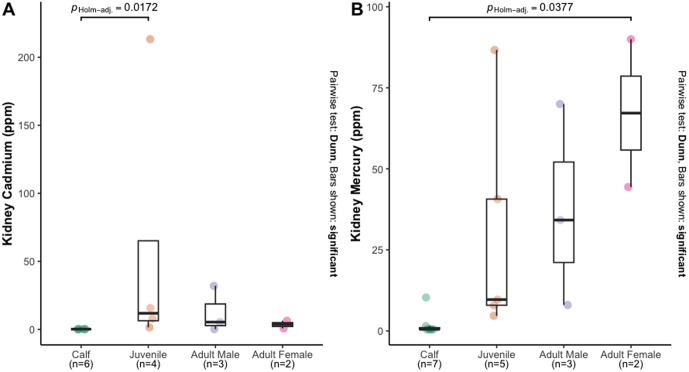
Box and whisker plots of elements from kidney based on life stage and sex (adults only) for (A) cadmium and (B) mercury. Boxes represent the 1st and 3rd quartiles, middle line is the median, and whiskers are 1.5 times the interquartile range. Jitter data points are denoted by circles. Statistical significance from the Kruskal-Wallis rank sum test and post hoc Dunn’s test with a Holm correction are indicated above in brackets.

**Fig 4:**
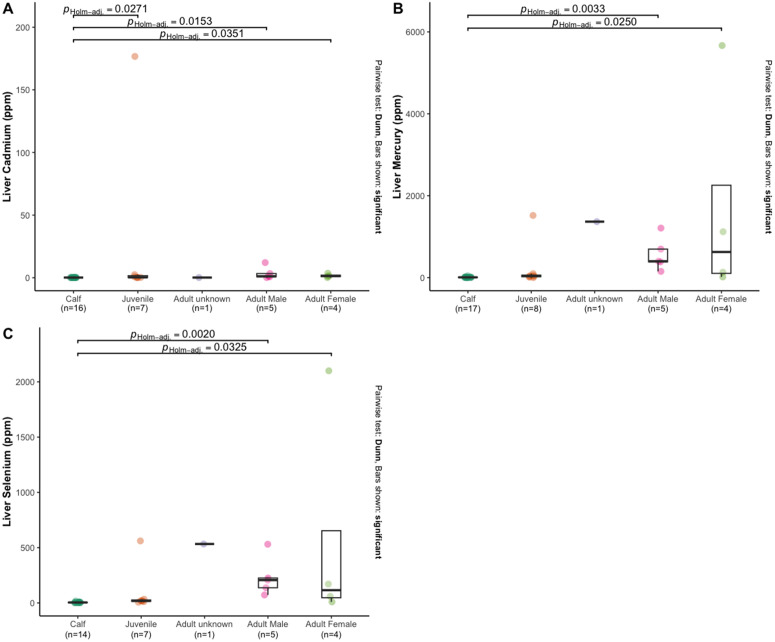
Box and whisker plots of elements from liver based on life stage and sex (adults only) for (A) cadmium, (B) mercury, and (C) selenium. Boxes represent the 1st and 3rd quartiles, middle line is the median, and whiskers are 1.5 times the interquartile range. Jitter data points are denoted by circles. Statistical significance from the Kruskal-Wallis rank sum test and post hoc Dunn’s test with a Holm correction are indicated above in brackets.

**Fig 5:**
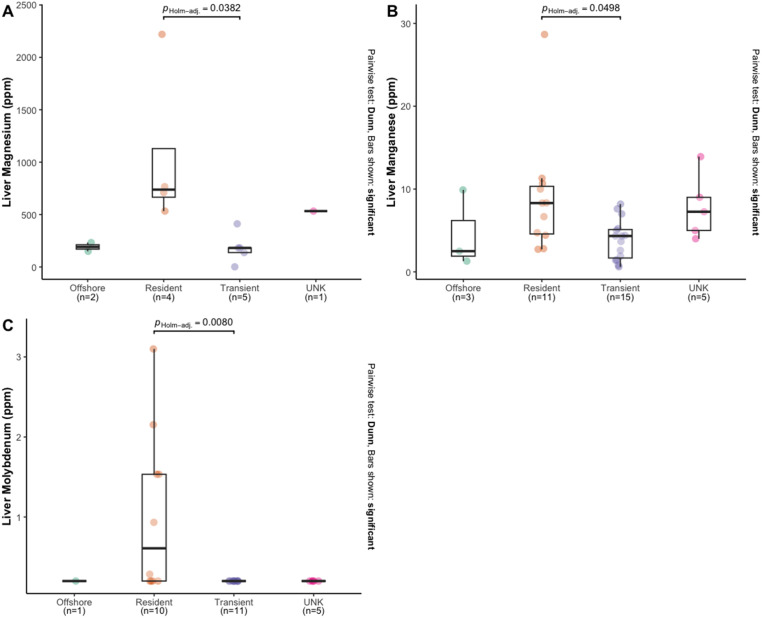
Box and whisker plots of elements from liver based on ecotype for (A) magnesium, (B) manganese, and (C) molybdenum. Boxes represent the 1st and 3rd quartiles, middle line is the median, and whiskers are 1.5 times the interquartile range. Jitter data points are denoted by circles. Statistical significance from the Kruskal-Wallis rank sum test and post hoc Dunn’s test with a Holm correction are indicated above in brackets.

Pairwise comparisons using the Kruskal-Wallis rank sum test showed kidney Se concentrations differed by life stage (p = 0.0307, ε^2^ = 0.556), however post hoc Dunn’s test did not detect statistically significant differences which likely reflect limited statistical power (p > 0.05). There were significant differences in Cd and tHg concentrations from kidney based on group life stage medians (p = 0.0205, ε^2^ = 0.612 and p = 0.0138, ε^2^ = 0.666, respectively). On average juveniles (n = 4; median = 11.8 ppm) had higher Cd concentrations than calves (n = 6; median = 0.150 ppm; p = 0.0172; S1 Table; Fig 3A). Also, adult females (n = 2; median = 67.2 ppm) had higher tHg concentrations than calves (n = 7; median = 0.500 ppm; p = 0.0377; S1 Table; Fig 3B). Pairwise comparisons in liver based on life stage showed significant differences in Cu, Mn, and MeHg concentrations (p = 0.049, ε^2^ = 0.280; p = 0.0443, ε^2^ = 0.288; and p = 0.039, ε^2^ = 0.297, respectively), however post hoc Dunn’s test did not detect statistically significant differences which likely reflect limited statistical power (p > 0.05). There were significant differences in group life stage medians for liver Cd (p = 0.0010, ε^2^ = 0.541) with higher concentrations in adult females (n = 4; median = 1.33 ppm; p = 0.0351), adult males (n = 5; median = 1.15 ppm; p = 0.0153), and juveniles (n = 7; median = 0.300 ppm; p = 0.0271) than calves (n = 16; median = 0.150 ppm; S1 Table; Fig 4A). There were also significant differences in group life stage medians for liver tHg (p < 0.001, ε^2^ = 0.623) with higher concentrations in adult females (n = 4; median = 626 ppm; p = 0.0250) and adult males (n = 5; median = 400 ppm; p = 0.0033) than calves (n = 17; median = 6.77 ppm; S1 Table; Fig 4B). Significant differences in group life stage medians for liver Se (p < 0.001, ε^2^ = 0.639) showed higher concentrations in adult females (n = 4; median = 115 ppm; p = 0.0325) and adult males (n = 5; median = 207 ppm; p = 0.0020) than in calves (n = 14; median = 4.32 ppm; S2 Table; Fig 4C). Pairwise comparisons based on ecotype also showed significant differences in median concentrations from liver Mg, Mn, and Mo (p = 0.0383, ε^2^ = 0.247; p = 0.0277, ε^2^ = 0.268; and p = 0.0062, ε^2^ = 0.364, respectively). On average, residents (n = 4; median = 738 ppm) had higher Mg concentrations than transients (n = 5; median = 182 ppm; p = 0.0382; S2 Table; Fig 5A). Residents also had higher Mn concentrations (n = 11; median = 8.31 ppm) than did transient killer whales (n = 15; median = 4.33 ppm; p = 0.0498; S2 Table; Fig 5B), and residents had higher Mo concentrations (n = 10; median = 0.609 ppm) than transient killer whales (n = 11; median = 0.200 ppm; p = 0.0080; S2 Table; Fig 5C).

In the GLM, regression of log (tHg) concentrations from kidney in response to explanatory variables found AIC score favored a model including life stage (57.9) over an intercept-only model (75.0) by a difference of a change in AIC > 2. On average, the expected tHg concentration is lower in calves compared to other life stages (4.25 ppm; GLM *b* = 4.25, p < 0.001; S1 Fig). The model explains 74.3% of the variability in kidney tHg concentrations when accounting for life stage. Regression of log (Mg) concentrations from liver in response to explanatory variables found AIC score favored a model including population and life stage (17.3) over an intercept-only model (50.3) by a difference of a change in AIC > 2. On average, the expected Mg concentration is lower in Transient (NT1 haplotype) and WCT killer whales compared to other populations (4.70 ppm, GLM *b* = 4.70, p = 0.0376 and 5.80 ppm, GLM *b* = 5.80, p = 0.0118, respectively; S2 Fig), whereas the expected Mg concentration is higher in adult male killer whales (4.22 ppm; GLM *b* = 4.22, p = 0.0247; S2 Fig) compared to other life stages. The model explains 98.6% of the variability in liver Mg concentrations when accounting for population and life stage. Regression of log (Mn) concentrations from liver in response to explanatory variables found AIC score favored a model including ecotype and life stage (74.0) over an intercept-only model (87.4) by a difference of a change in AIC > 2. On average, the expected Mn concentration is higher in adults of unknown sex, calves, and juvenile killer whales compared to other life stages (1.63 ppm, GLM *b* = 1.63, p = 0.0318; 1.06 ppm, GLM *b* = 1.06, p = 0.0066; and 1.15 ppm, GLM *b* = 1.15, p = 0.0107, respectively; S2 Fig). The model explains 55.3% of the variability in liver Mn concentrations when accounting for ecotype and life stage. Regression of log (Mo) concentrations from liver in response to explanatory variables found AIC score favored a model including ecotype and life stage (59.4) over an intercept-only model (71.7) by a difference of a change in AIC > 2. On average, the expected Mo concentration is lower in calves compared to other life stages (1.22 ppm; GLM *b* = 1.22, p = 0.0199; S2 Fig). The model explains 62.2% of the variability in liver Mo concentrations when accounting for ecotype and life stage. Regression of log (Cd) from liver in response to explanatory variables found AIC score favored a model including population and life stage (95.6) over an intercept-only model (131) by a difference of a change in AIC > 2. On average, the expected Cd concentration is higher in Resident (unspecified) and Transient (NT1 haplotype) killer whales compared to other populations (6.71 ppm, GLM *b* = 6.71, p < 0.0001 and 3.21 ppm, GLM *b* = 3.21, p = 0.0297, respectively; S2 Fig), whereas the expected Cd concentration is lower in calves (1.79 ppm; GLM *b* = 1.79, p = 0.0023) compared to other life stages. The model explains 84.3% of the variability in liver Cd concentrations when accounting for population and life stage. Regression of log (tHg) concentrations from liver in response to explanatory variables found AIC score favored a model including life stage and sex (148) over an intercept-only model (168) by a difference of a change in AIC > 2. On average, the expected tHg concentration is lower in calves and juvenile killer whales compared to other life stages (4.60 ppm, GLM *b* = 4.60, p = 0.0017 and 3.28 ppm, GLM *b* = 3.28, p = 0.0081, respectively; S2 Fig). The model explains 60.0% of the variability in liver tHg concentrations when accounting for life stage and sex. Regression of log (Se) concentrations from liver in response to explanatory variables found AIC score favored a model including life stage (106) over an intercept-only model (134) by a difference of a change in AIC > 2. On average, the expected Se concentration is lower in calves compared to other life stages (3.35 ppm, GLM *b* = 3.35, p < 0.0001; S2 Fig). The model explains 69.2% of the variability in liver Se concentrations when accounting for life stage.

## Discussion

Relatively little is known about elements and their potential effects on killer whales in the eastern North Pacific and these findings significantly expand our knowledge of essential and non-essential elements in killer whales and build on prior research that obtained elemental concentrations from fewer numbers of stranded killer whales in other parts of the world [5,6,18,31–33]. In a rare case, Endo et al. [32,33] examined a small group of killer whales that stranded together in northern Japan. Although the sample size was small (n = 9), it was believed that the stranded individuals were genetically related and more than likely shared the same dietary and spatial habits based on their stomach contents. Our study was a retrospective analysis using opportunistically collected samples over time. While this prohibits us from understanding some of the nuanced details of how elements accumulate in killer whales, it more than doubled the number of killer whales sampled and analyzed for elements to date [32,33,61]. Investigating disease and determining elemental concentrations in killer whales can be challenging given the rarity of killer whale strandings in the Northeastern Pacific Ocean [62]. Despite the small sample size, this represents one of the largest such datasets globally due to stranding rarity. Postmortem investigations found that no animals examined had microscopic lesions consistent with elevated element concentrations [43]. We cannot, however, be certain if any of these element concentrations had more obscure reproductive, subclinical, or immunotoxic effects on animals prior to stranding. Therefore, the concentrations we describe should provide a starting point for better understanding the association between elements and killer whale health in the eastern North Pacific Ocean.

### Cadmium

The average concentration of Cd varied by tissue, with kidney having almost three times the concentration detected in liver. This is consistent with research showing that Cd accumulates in the kidneys of other cetaceans [5,24,63,64]. We found kidney Cd concentrations were higher in juveniles than in calves. We also found liver Cd concentrations were higher in adult females, adult males, and juveniles than in calves. This corroborates with Endo et al. [33] who found that liver Cd concentrations in killer whales in northern Japan increased with age. Age-related trends were less certain for kidney Cd concentrations. It is possible that Cd concentrations in kidney could plateau at some point, as it did in striped dolphins [65]. Despite the small sample sizes which may limit statistical power, our model also suggests, with relatively high probability, that population and life stage may have some effect on liver Cd concentrations with Cd expected to be higher in unspecified residents and NT1 haplotype transients than other populations, and that Cd concentrations are lower in calves than in other life stages. The similar trends seen between our work and those from Endo et al. [33] could be related to sample size, or Cd availability could be spatially similar in the NE Pacific, the NW Pacific, and the northern waters off Japan. Nutrient upwellings due to shifting El Niño and La Niña events could also be a source of environmental contamination of Cd [66–69]. Variability in concentrations could be related to differences in dietary concentrations in prey items or bioavailability and sediment concentrations [70,71]. The stomach contents of killer whales stranded off the northern coast of Japan included remnants of seal and squid tissue. Higher Cd concentrations in those older animals makes sense, given that Cd is known to concentrate in cephalopods and accumulates in odontocetes through diet and over a lifetime [3,10,64,72–79]. Previous research has demonstrated that Cd can transfer via breast milk and gestation in humans [80–82], but not in some pinnipeds [37,83]. It is not known if offloading of Cd occurs in killer whales or other cetaceans through lactation or in utero, but the low concentrations found in calves in this study may be related to maternal transfer.

### Mercury

There were twice as many liver samples analyzed for tHg than kidney, and average concentrations varied by tissue type. Historic measurement of tHg concentrations uses liver, but tHg is found in other tissues like brain, kidney, and muscle [84]. We found that liver from adult females and adult males had higher concentrations of tHg than calves. Our model also suggests, with some uncertainty, that life stage and sex may have some effect on liver tHg concentrations with tHg expected to be lower in calves and juveniles. These results corroborate with Endo et al. [33], who found higher concentrations in the liver of mature killer whales than in calves. Exposure to Hg is often through diet and the little difference between the calf and juvenile life stage could be that juveniles in our data may have been in the transition period of foraging for food, but not fully weaned [84]. While MeHg in liver differed by life stage, the fact that no groups were found statistically different indicate there might be another factor influencing the concentration of MeHg in these whales like differences in dietary sources [85]. Age accumulation of Hg is also evident in other marine mammals, including bottlenose dolphins, pygmy and dwarf (*Kogia sima*) sperm whales, long-finned pilot whales (*Globicephala melas edwardii*), Pacific harbor seals (*Phoca vitulina richardii*), common dolphins, beluga whales, and various cetaceans in the Pacific Islands [5,7,13,24,64,78,86]. This general increasing trend makes sense given that tHg biomagnifies and accumulates over time [5,25]. Another source for accumulation could be delivered through nutrient upwellings [87]. It may be worth exploring the subclinical reproductive effects of high tHg in endangered SRKW females.

### Selenium

Concentration of Se varied by tissue type. We found on average, liver from adult females and adult males had higher Se concentrations than calves. The model also suggests, with some confidence, that life stage may have some effect on liver Se with lower concentrations expected in calves. This corroborates with several other studies. For example, Endo et al. [32] showed mature female killer whales had higher Se concentrations than calves, but the concentrations from our study were markedly higher overall. This difference could be because concentrations of some elements vary by what is naturally or not naturally occurring [64]. Similarly, a collection of cetaceans stranded in the U.S. Pacific Islands also showed higher Se concentrations in older animals [5], as did harbor seals from the inland waters of Washington State [88]. Another study showing differences based on age class identified higher Se concentrations in adult pygmy sperm whales than in calves and subadults [21]. Liver Se concentrations in long-finned pilot whales also were higher the larger the animal [64].

### Relationship between mercury and selenium

The relationship between Hg and Se is well known in marine mammals, and the strong correlation observed between these two elements was initially observed in the liver by Koeman et al. [89]. Extensive research in other marine mammals including various cetaceans [6] and Guiana dolphins on the coast of Brazil [2], cetaceans in the U.S. Pacific Islands [5], Pacific harbor seals in San Juan County [7], and killer whales on the coast of Japan [32] showed this tight relationship. We, too, found that kidney and liver Se and Hg are strongly correlated. Between the two tissue types, the liver Se:Hg relationship is a slightly better fit than in kidney. We also found a positive relationship between Se and MeHg in the liver, but not in the kidney and that may be from the small sample size given that liver had more than three times as many pairs. Mercury is an extremely toxic element that can be neurotoxic, immunotoxic, hepatotoxic, nephrotoxic, and toxic to developing fetuses [13,16,17,84,90,91]. The liver counters the harmful effects of Hg through demethylation and sequestration by cysteine-rich binding proteins called metallothioneins [73,92,93]. Another important mechanism for detoxification is the formation of non-bioavailable Se:Hg complexes in the liver [5,10,21,84,89]. When Se is less readily bioavailable to bind with Hg, free Hg can promote free radical formation causing oxidative stress [93]. In NE Pacific killer whales, we found that Hg and Se concentrations had similar trends and increased with age. The average Se:Hg molar ratios in this study were nearly or just above 1:1 in both kidney and liver. These corroborate with Se:Hg molar ratios found in the liver (mean = 1396 ppm dw, molar ratio = 1.06) and kidney (mean = 945 ppm dw, molar ratio = 0.86) of killer whales collected for human consumption off St. Vincent, West Indies despite having some of the highest reported mean Hg concentrations in killer whales [61]. When considering molar ratios based on variables, we found in general similar 1:1 trends in the liver, but a high variability in molar ratios in the kidney. We are unsure if this variability is due to fewer sample sizes available or because of the nuances in the interactions that occurs between the two elements varying in different tissues. Overall, the mean Se:Hg molar ratios in this study seems consistent with prior research in other marine mammals (liver ratio 1:1 [89]; liver ratio averaging 1.2 [2]; ratio ranging from 0.24 to 0.90 in the kidney and from 0.81 to 1.19 in the liver [32]; liver ratio ranging from 0.009–1.039 [21]; liver ratio ranging from 0.94 to 1.8 [6]; liver ratio ranging from 0.003 to 1.03 [5]; liver ratio averaging 1.36 [7]; ratio ranging from 1.57 to 5.72 in the kidney and from 0.895 to 4.72 in the liver [94]). Although this study does not measure oxidative stress indicators or metallothionein expression, some consider Se:Hg molar ratios as protective which highlight that sufficient Se protection may have prevented or reduced the risk of Hg toxicosis for the killer whales we sampled [5,10,92]. However, protective effects are speculative without direct biomarkers (*e.g.*, oxidative stress, metallothioneins).

### Magnesium

Far fewer kidney samples were analyzed for Mg than were liver, so it is difficult to compare concentrations by tissue. We found one difference in liver Mg based on ecotype, with resident killer whales having higher concentrations than transients. Despite the small sample sizes which may limit statistical power, our model also suggests, with relatively high probability, that population and life stage may have some effect on Mg, with transients from the NT1 haplotype and WCT killer whales expected to have lower Mg concentrations. Also, adult males were expected to have higher concentrations. Magnesium is an essential element homeostatically regulated for muscle movement and is known to have antioxidant effects [3,95,96]. A few studies have analyzed Mg concentrations in tissues of odontocetes [3,97–102], but little is known about the factors that affect concentrations.

### Manganese

Manganese is an essential element required for some developmental and physiologic functions [103]. As for other elements, fewer kidney samples were analyzed for Mn than were liver, so it is difficult to compare concentrations by tissue. We found liver Mn concentrations varied by ecotype, with resident killer whales having higher concentrations than transients. This could be related to ecotype-specific differences in energetic and metabolic demands [104–107]. Manganese naturally occurs in marine sediments and becomes more bioavailable under redox conditions [108]. However, it is not known if Mn concentrations in prey increase through trophic transfer. Our model suggests, with some uncertainty, that liver Mn concentration varies by ecotype and life stage with calves, juveniles, and adults with unknown sex expected to have higher concentrations than other life stages. The uncertainty in our model may be due to the small sample size in each life stage. Metabolic requirements in an individual’s life history, as well as sex-related differences, could affect tissue Mn concentrations [23]. Much like killer whales, maturation rates in odontocetes differ between males and females, with females reaching sexual maturity earlier than males [109–111]. Previous research by Monteiro et al. [23] corroborates those relationships. The authors identified sex and body length (a proxy for age) had some effect on Mn concentrations in the liver of bottlenose dolphins.

### Molybdenum

Molybdenum is a naturally occurring essential element that is typically ingested [112]. Average Mo concentrations varied, but there were twice as many liver samples analyzed for Mo than kidney, so it is difficult to compare concentration differences between tissue types. Studies have measured Mo concentrations in cetaceans, but few have identified the factors affecting concentration. A study on small cetaceans along the Brazilian coast found that liver Mo concentrations in Guiana dolphins and franciscana dolphins increased with age, with no differences between the species [2]. Another study showed species-specific differences with higher liver Mo concentrations in franciscana dolphins than Guiana dolphins and showed sex and age-related differences in franciscana dolphins (higher concentrations in females than males and an increase in concentrations with age) [79]. We found resident killer whales had higher liver Mo concentrations than did transients. Our model also suggests, with some level of uncertainty, that ecotype and life stage may have some effect on liver Mo concentrations, with lower concentrations expected in calves. The differences between killer whale ecotype and life stage may be related to their contrasting diet and foraging techniques. It is not known if this element is concentrated in odontocetes by trophic transfer.

### Measuring contaminants in kidney vs. liver

Elemental concentrations in marine mammals have been measured in a variety of tissues. For example, Endo et al. [32,33] used liver, kidney, and muscle to measure elements in killer whales. Cáceres-Saez et al. [91] tested elements in liver, kidney, spleen, lung, skeletal muscle, reproductive organs, and skin from subantarctic false killer whales (*Pseudorca crassidens*). Page et al. [24] also reviewed tissue-specific differences in elemental concentrations using blubber, feces, kidney, liver, skeletal muscle, and skin samples from various odontocetes. Elemental concentrations vary by study. For example, liver Se concentrations were higher than in the kidney or muscle of killer whales [32]. Cáceres-Saez et al. [91] found higher Se concentrations in the skin and liver of false killer whales. Differences in sample storage, preparation, analysis, and species can make it difficult to compare contaminant concentrations between tissues and across multiple studies. Further research is needed to better understand how elements accumulate in odontocetes and how concentrations vary by tissue type. We had half as many kidney samples as we did liver, which often made it difficult to compare contaminant concentrations by tissue type. We suggest future killer whale necropsies sample liver and kidney for essential and non-essential elements to better understand differences in tissue concentrations.

## Conclusions

This work provides novel data to improve our understanding of elements in killer whales from the eastern North Pacific. Variability in elemental concentrations by life stage, sex, ecotype, and population is likely influenced by differing spatial use, diet, foraging techniques, bioavailability, environmental sources from upwelling and sediment concentrations, as well as energetic and metabolic demands. Furthermore, these baseline data can inform future biomonitoring and threat assessment for other endangered populations. Future work will help us better understand the role elements play in affecting killer whale health, especially in the endangered SRKW population.

## Supporting information

S1 Table Summary of mean  ±  standard deviation (SD), median, and sample size (n) shown in parenthesis for non-essential elements from kidney and liver separated based on sex, life stage, ecotype, and population. Values are reported in ppm dw unless otherwise noted.
(DOCX)

S2 Table Summary of mean  ±  standard deviation, median, and sample size (n) shown in parenthesis for essential elements from kidney and liver separated based on sex, life stage, ecotype, and population. Values are reported in ppm dw.
(DOCX)

S1 Fig Diagnostic plots for kidney GLM. (DOCX)

S2 Fig Diagnostic plots for liver GLMs. (DOCX)
