# Supplementary material for: Concentrations of essential and non-essential elements in eastern North Pacific killer whales (Orcinus orca)
Source: PLoS One. 2026 Jul 15;21(7):e0353196. doi: 10.1371/journal.pone.0353196 (PMC13372180; doi:10.1371/journal.pone.0353196)
Supplement: S1 Table — Values are reported in ppm dw unless otherwise noted. (DOCX) [file pone.0353196.s001.docx]

|  | **Tissue** | **As ± SD, median,**  **(n)** | **Ba ± SD, median, (n)** | **Be ± SD, median, (n)** | **Cd ± SD, median (n)** | **Cr ± SD, median, (n)** | **Pb ± SD, median, (n)** | **Hg ± SD, median, (n)** | **MeHg* ± SD, median, (n)** | **Ni ± SD, median, (n)** | **Tl ± SD, median, (n)** |
| --- | --- | --- | --- | --- | --- | --- | --- | --- | --- | --- | --- |
| **Sex** | | | | | | | | | | | |
| Female | kidney | 0.552±  0.123,  0.500 (7) | 0.0250,  0.0250 (1) | 0.0250,  0.0250 (1) | 2.41±3.30,  0.700 (7) | 0.0250,  0.0250 (1) | 0.366±  0.228,  0.500 (7) | 22.2±30.6,  7.93 (9) | 1757±  2811,  270 (3) | 0.203±0.251,  0.203 (2) | 0.0500,  0.0500 (1) |
|  | liver | 0.539±  0.0668,  0.500 (12) | 0.0490±  0.0160,  0.0500 (5) | 0.0139±  0.0065,  0.0100 (5) | 0.610±  0.970,  0.150 (14) | 0.280±  0.255,  0.150 (5) | 0.572±  0.273,  0.500 (14) | 446±1419,  11.4 (16) | 4044±  4826,  2259 (10) | 0.314±  0.0863,  0.300 (6) | 0.450± 0.240,  0.500 (5) |
| Male | kidney | 1.26±2.09,  0.500 (7) | 0.136±  0.157,  0.136 (2) | 0.0250±0,  0.0250 (2) | 38.1±78.1,  5.33 (7) | 0.636±  0.892,  0.636 (2) | 0.364±  0.232,  0.500 (7) | 29.9±35.3,  9.67 (7) | 9983±  11809,  9983 (2) | 0.713±0.972,  0.713 (2) | 0.0375±  0.0177,  0.0375 (2) |
|  | liver | 0.631±  0.490,  0.500 (14) | 0.0475±  0.0079,  0.0500 (10) | 0.0115±  0.0047, 0.0100 (10) | 12.4±43.9, 0.150 (16) | 0.306±  0.547,  0.150 (10) | 0.533±  0.217,  0.500 (16) | 285±468,  43.3 (16) | 5423±  8354,  1179 (9) | 0.273±  0.0870,  0.300 (10) | 0.453± 0.150,  0.500 (10) |
| Unknown Sex | kidney | 0.203,  0.203 (1) | 0.0250,  0.0250 (1) | 0.0250,  0.0250 (1) | 0.0250,  0.0250 (1) | 0.0250,  0.0250 (1) | 0.0250,  0.0250 (1) | 1.50,  1.50 (1) | 333,  333 (1) | 0.0250,  0.0250 (1) | 0.0500,  0.0500 (1) |
|  | liver | 0.500±0,  0.500 (2) | 0.0500±0,  0.0500 (2) | 0.0100±0,  0.0100 (2) | 0.133±  0.0289,  0.150 (3) | 0.150±0, 0.150 (2) | 0.667±  0.289,  0.500 (3) | 459±786,  9.79 (3) | 7373±  11803,  733 (3) | 0.300±0,  0.300 (2) | 0.500±0,  0.500 (2) |
| **Life Stage** | | | | | | | | | | | |
| Adult Female | kidney | 0.533,  0.533 (1) | 0.0250,  0.0250 (1) | 0.0250,  0.0250 (1) | 3.52±3.98,  3.52 (2) | 0.0250,  0.0250 (1) | 0.0250,  0.0250 (1) | 67.2±32.2,  67.2 (2) | 5000,  5000 (1) | 0.0250,  0.0250 (1) | 0.0500,  0.0500 (1) |
|  | liver | 0.567±  0.0943,  0.567 (2) | 0.0375±  0.0177,  0.0375 (2) | 0.0175±  0.0106,  0.0175 (2) | 1.60±1.45,  1.33 (4) | 0.442±  0.412,  0.442 (2) | 0.578±  0.389,  0.500 (3) | 1734±2668,  626 (4) | 9721±  8880,  9721 (2) | 0.238±  0.0872,  0.238 (2) | 0.275±  0.318,  0.275 (2) |
| Adult Male | kidney | 2.33±3.18,  0.500 (3) | - | - | 12.5±17.1,  5.33 (3) | - | 0.500±0,  0.500 (3) | 37.4±31.1,  34.2 (3) | 1633,  1633 (1) | - | - |
|  | liver | 0.500±0,  0.500 (4) | 0.0500±0,  0.0500 (2) | 0.0100±0,  0.0100 (2) | 3.51±4.94,  1.15 (5) | 0.150±0, 0.150 (2) | 0.600±  0.224,  0.500 (5) | 568±407,  400 (5) | 5714±  1629,  5900 (3) | 0.300±0,  0.300 (2) | 0.500±0,  0.500 (2) |
| Adult Unknown | kidney | - | - | - | - | - | - | - | - | - | - |
|  | liver | 0.500,  0.500 (1) | 0.0500,  0.0500 (1) | 0.0100,  0.0100 (1) | 0.150,  0.150 (1) | 0.150,  0.150 (1) | 0.500,  0.500 (1) | 1367,  1367 (1) | 21000,  21000 (1) | 0.300,  0.300 (1) | 0.500,  0.500 (1) |
| Juvenile | kidney | 0.538±  0.253,  0.550 (4) | 0.136±  0.157,  0.136 (2) | 0.0250±0,  0.0250 (2) | 59.6±103,  11.8 (4) | 0.636±  0.892,  0.636 (2) | 0.147±  0.235,  0.0318 (4) | 29.9±34.9,  9.67 (5) | 6202±  10507,  270 (3) | 0.602±0.714,  0.380 (3) | 0.0375±  0.0177,  0.0375 (2) |
|  | liver | 0.849±  0.731,  0.530 (6) | 0.0490±  0.0160,  0.0500 (5) | 0.0139±  0.0065,  0.0100 (5) | 25.8±66.5, 0.300 (7) | 0.134±  0.0774, 0.150 (5) | 0.472±  0.339,  0.500 (7) | 222±524,  39.7 (8) | 7637±  11074,  2551 (5) | 0.289±0.140,  0.300 (6) | 0.445 ±  0.250,  0.500 (5) |
| Calf | kidney | 0.458±  0.112,  0.500 (7) | 0.0250,  0.0250 (1) | 0.0250,  0.0250 (1) | 0.129±  0.0510,  0.150 (6) | 0.0250,  0.0250 (1) | 0.432±  0.180,  0.500 (7) | 2.05±3.67,  0.500 (7) | 333,  333 (1) | 0.0250,  0.0250 (1) | 0.0500,  0.0500 (1) |
|  | liver | 0.505± 0.0207,  0.500 (15) | 0.0500±0,  0.0500 (7) | 0.0100±0,  0.0100 (7) | 0.141±  0.0202,  0.150 (16) | 0.394±  0.646,  0.150 (7) | 0.588±  0.196,  0.500 (17) | 9.46±10.4,  6.77 (17) | 1418±  1468,  1024 (11) | 0.300±0,  0.300 (7) | 0.500±0,  0.500 (7) |
| **Ecotype** | | | | | | | | | | | |
| Offshore | kidney | - | - | - | - | - | - | - | - | - | - |
|  | liver | 0.540±  0.0566,  0.540 (2) | 0.0500,  0.0500 (1) | 0.0100,  0.0100 (1) | 1.28±2.01, 0.150 (3) | 0.150,  0.150 (1) | 0.833±  0.289,  1.00 (3) | 45.9±74.6,  4.10 (3) | 1024,  1024 (1) | 0.300,  0.300 (1) | 0.500,  0.500 (1) |
| Resident | kidney | 1.31±2.08, 0.533 (7) | 0.0250±0,  0.0250 (3) | 0.0250±0,  0.0250 (3) | 33.3±79.4,  5.33 (7) | 0.0183±  0.0115,  0.0250 (3) | 0.231±  0.252,  0.0387 (7) | 28.5±34.6,  8.83 (8) | 1742±  2283,  983 (4) | 0.114±0.178,  0.0250 (4) | 0.0417±  0.0144,  0.0500 (3) |
|  | liver | 0.723±  0.570,  0.500 (10) | 0.0463±  0.0148,  0.0500 (8) | 0.0143±  0.0068,  0.0100 (8) | 16.5±53.1, 0.150 (11) | 0.426±  0.618,  0.150 (8) | 0.458±  0.273,  0.500 (11) | 624±1626,  23.6 (12) | 3738±  5055,  1065 (10) | 0.279±0.117,  0.300 (9) | 0.409±  0.240,  0.500 (8) |
| Transient | kidney | 0.500±0,  0.500 (3) | - | - | 8.24±15.8,  0.425 (4) | - | 0.500±0,  0.500 (3) | 19.9±22.8,  17.4 (4) | - | - | - |
|  | liver | 0.500±0,  0.500 (11) | 0.0500±0,  0.0500 (6) | 0.0100±0,  0.0100 (6) | 1.28±3.11, 0.150 (15) | 0.150±0, 0.150 (6) | 0.607±  0.213,  0.500 (14) | 260±445,  25.0 (15) | 6608±  7457,  4554 (6) | 0.300±0,  0.300 (6) | 0.500±0,  0.500 (6) |
| Unknown Ecotype | kidney | 0.444±  0.125,  0.500 (5) | 0.247,  0.247 (1) | 0.0250,  0.0250 (1) | 4.35±7.57,  0.792 (4) | 1.27,  1.27 (1) | 0.405±  0.212,  0.500 (5) | 20.5±37.2,  4.67 (5) | 9302±  12773,  9302 (2) | 1.40,  1.40 (1) | 0.0500,  0.0500 (1) |
|  | liver | 0.500±0,  0.500 (5) | 0.0500±0,  0.0500 (2) | 0.0100±0,  0.0100 (2) | 0.723±1.15, 0.150 (4) | 0.150±0, 0.150 (2) | 0.500±0,  0.500 (5) | 310±675,  9.33 (5) | 6663±  11238,  1967 (5) | 0.300±0,  0.300 (2) | 0.500±0,  0.500 (2) |
| **Population** | | | | | | | | | | | |
| Alaska Resident | kidney | - | - | - | - | - | - | - | - | - | - |
|  | liver | 0.700,  0.700 (1) | 0.0700,  0.0700 (1) | 0.0145,  0.0145 (1) | 0.215,  0.215 (1) | 0.215,  0.215 (1) | 0.700,  0.700 (1) | 96.7,  96.7 (1) | 7721,  7721 (1) | 0.430,  0.430 (1) | 0.700,  0.700 (1) |
| Southern Resident | kidney | 1.88±2.76, 0.665 (4) | 0.0250,  0.0250 (1) | 0.0250,  0.0250 (1) | 3.37±3.94,  2.74 (4) | 0.0250,  0.0250 (1) | 0.266±  0.270,  0.269 (4) | 21.9±32.2,  7.97 (4) | 656±862,  333 (3) | 0.203±0.251,  0.203 (2) | 0.0500,  0.0500 (1) |
|  | liver | 0.512±  0.0268,  0.500 (5) | 0.0500±0,  0.0500 (3) | 0.0100±0,  0.0100 (3) | 0.419±  0.449,  0.150 (6) | 0.719±  0.986,  0.150 (3) | 0.513±  0.293,  0.500 (6) | 277±482,  23.6 (6) | 1921±  2433,  620 (6) | 0.320±  0.0400,  0.300 (4) | 0.500±0,  0.500 (3) |
| Northern Resident | kidney | 0.517±  0.0236, 0.517 (2) | 0.0250,  0.0250 (1) | 0.0250,  0.0250 (1) | 3.24±4.37,  3.24 (2) | 0.0250,  0.0250 (1) | 0.263±  0.336,  0.263 (2) | 43.7±44.8,  40.7 (3) | 5000,  5000 (1) | 0.0250,  0.0250 (1) | 0.0500,  0.0500 (1) |
|  | liver | 0.544±  0.0770,  0.500 (3) | 0.0417±  0.0144,  0.0500 (3) | 0.0150±  0.0087,  0.0100 (3) | 0.589±  0.760,  0.150 (3) | 0.344±  0.337,  0.150 (3) | 0.411±  0.154,  0.500 (3) | 1420±2831,  7.47 (4) | 6043±  8623,  1138 (3) | 0.259±  0.0712,  0.300 (3) | 0.350±  0.260,  0.500 (3) |
| Resident  (unspecified) | kidney | 0.600,  0.600 (1) | 0.0250,  0.0250 (1) | 0.0250,  0.0250 (1) | 213,  213 (1) | 0.0050,  0.0050 (1) | 0.0250,  0.0250 (1) | 9.67,  9.67 (1) | - | 0.0250,  0.0250 (1) | 0.0250,  0.0250 (1) |
|  | liver | 2.33,  2.33 (1) | 0.0250,  0.0250 (1) | 0.0250,  0.0250 (1) | 177,  177 (1) | 0.0050,  0.0050 (1) | 0.0250,  0.0250 (1) | 46.7,  46.7 (1) | - | 0.0250,  0.0250 (1) | 0.0250,  0.0250 (1) |
| Offshore | kidney | - | - | - | - | - | - | - | - | - | - |
|  | liver | 0.540±  0.0566,  0.540 (2) | 0.0500,  0.0500 (1) | 0.0100,  0.0100 (1) | 1.28±2.01, 0.150 (3) | 0.150,  0.150 (1) | 0.833±  0.289,  1.00 (3) | 45.9±74.6,  4.10 (3) | 1024,  1024 (1) | 0.300,  0.300 (1) | 0.500,  0.500 (1) |
| Gulf of Alaska Transient | kidney | - | - | - | - | - | - | - | - | - | - |
|  | liver | 0.500±0,  0.500 (2) | 0.0500,  0.0500 (1) | 0.0100,  0.0100 (1) | 0.150±0, 0.150 (2) | 0.150,  0.150 (1) | 0.500±0,  0.500 (2) | 694±951,  694 (2) | 13333±  10842,  13333 (2) | 0.300,  0.300 (1) | 0.500,  0.500 (1) |
| West Coast Transient | kidney | 0.500±0,  0.500 (2) | - | - | 0.150±0,  0.150 (2) | - | 0.500±0,  0.500 (2) | 0.500±0,  0.500 (2) | - | - | - |
|  | liver | 0.500±0,  0.500 (6) | 0.0500±0,  0.0500 (3) | 0.0100±0,  0.0100 (3) | 0.225±  0.233,  0.150 (8) | 0.150±0, 0.150 (3) | 0.625±  0.231,  0.500 (8) | 30.7±50.6,  12.3 (8) | 2310±  1600,  2310 (2) | 0.300±0,  0.300 (3) | 0.500±0,  0.500 (3) |
| Transient NT1 haplotype | kidney | 0.500,  0.500 (1) | - | - | 32.0,  32.0 (1) | - | 0.500,  0.500 (1) | 34.2,  34.2 (1) | - | - | - |
|  | liver | 0.500,  0.500 (1) | - | - | 12.1,  12.1 (1) | - | 0.500,  0.500 (1) | 385,  385 (1) | - | - | - |
| Transient  (unspecified) | kidney | - | - | - | 0.70,  0.700 (1) | - | - | 44.4,  44.4 (1) | - | - | - |
|  | liver | 0.500±0,  0.500 (2) | 0.0500±0,  0.0500 (2) | 0.0100±0,  0.0100 (2) | 1.25±1.47,  0.750  (4) | 0.150±0, 0.150 (2) | 0.667±  0.289,  0.500 (3) | 472±533,  371 (4) | 4181±  4330,  4181 (2) | 0.300±0,  0.300 (2) | 0.500±0,  0.500 (2) |
| Unknown Population | kidney | 0.444±  0.125,  0.500 (5) | 0.247,  0.247 (1) | 0.0250,  0.0250 (1) | 4.35±7.57,  0.792 (4) | 1.27,  1.27 (1) | 0.405±  0.212,  0.500 (5) | 20.5±37.2,  4.67 (5) | 9302±  12773,  9302 (2) | 1.40,  1.40 (1) | 0.0500,  0.0500 (1) |
|  | liver | 0.500±0,  0.500 (5) | 0.0500±0,  0.0500 (2) | 0.0100±0,  0.0100 (2) | 0.723±1.15, 0.150 (4) | 0.150±0, 0.150 (2) | 0.500±0,  0.500 (5) | 310±675,  9.33 (5) | 6663±  11238,  1967 (5) | 0.300±0,  0.300 (2) | 0.500±0,  0.500 (2) |

*parts per billion (ppb) dw
