# Supplementary material for: Concentrations of essential and non-essential elements in eastern North Pacific killer whales (Orcinus orca)
Source: PLoS One. 2026 Jul 15;21(7):e0353196. doi: 10.1371/journal.pone.0353196 (PMC13372180; doi:10.1371/journal.pone.0353196)
Supplement: S2 Table — Values are reported in ppm dw. (DOCX) [file pone.0353196.s002.docx]

|  | **Tissue** | **Ca ± SD, median, (n)** | **Co ± SD, median, (n)** | **Cu ± SD, median, (n)** | **Fe ± SD, median, (n)** | **Mg ± SD, median, (n)** | **Mn ± SD, median, (n)** | **Mo ± SD, median, (n)** | **Se ± SD, median, (n)** | **V ± SD, median, (n)** | **Zn ± SD, median, (n)** |
| --- | --- | --- | --- | --- | --- | --- | --- | --- | --- | --- | --- |
| **Sex** |  | | | | | | | | | | |
| Female | kidney | 2409±  2721,  2409 (2) | 0.0250,  0.0250 (1) | 11.0±  4.52,  10.8 (8) | 403±168,  383 (6) | 378±408,  378 (2) | 2.32±1.46,  2.03 (7) | 0.171±  0.0714,  0.200 (6) | 9.93±11.7,  5.45 (4) | 0.193,  0.193 (1) | 94.4±50.8,  86.7 (8) |
|  | liver | 91.1± 81.8,  76.5 (6) | 0.120±  0.0760, 0.150 (6) | 61.2±68.3,  50.0 (15) | 791±475,  833 (14) | 341±318,  208 (6) | 5.59±3.91,  5.00 (15) | 0.538±  0.877,  0.200 (11) | 173±556,  5.69 (14) | 0.479±  0.568,  0.215 (5) | 274±294,  113 (15) |
| Male | kidney | 551±430,  551(2) | 0.138±  0.159,  0.138 (2) | 10.8±  6.06,  8.92 (7) | 476±239,  467 (7) | 558±295,  558 (2) | 1.71±  0.605,  1.63 (7) | 0.152±  0.0818,  0.200 (7) | 9.08±8.64,  5.00 (7) | 0.403±  0.325,  0.403 (2) | 94.3±56.8,  70.0 (7) |
|  | liver | 256±179,  247 (5) | 0.138±  0.0395, 0.150 (10) | 82.8±82.4,  56.0 (16) | 657±329,  711 (16) | 360±190,  412 (5) | 5.48±3.14,  4.58 (16) | 0.530±  0.671,  0.200 (14) | 130±192,  18.6 (14) | 0.577±  0.563,  0.200 (10) | 278±222,  187 (16) |
| Unknown Sex | kidney | 467,  467 (1) | 0.0250,  0.0250 (1) | 25.3,  25.3 (1) | 600,  600 (1) | 367,  367 (1) | 1.27,  1.27 (1) | 0.0250,  0.0250 (1) | 3.07,  3.07 (1) | 0.180,  0.180 (1) | 113,  113 (1) |
|  | liver | 1727,  1727 (1) | 0.150±0, 0.150 (2) | 136±170,  43.3 (3) | 820±713,  433 (3) | 2220,  2220 (1) | 12.8±13.9,  7.00 (3) | 0.200±0,  0.200 (2) | 182±305,  9.44 (3) | 0.858±  1.00,  0.858 (2) | 269±145,  213 (3) |
| **Life Stage** |  | | | | | | | | | | |
| Adult Female | kidney | 2409±  2721,  2409 (2) | 0.0250,  0.0250 (1) | 9.73±8.39,  9.73 (2) | 400,  400 (1) | 378±408,  378 (2) | 1.37±  0.943,  1.37 (2) | 0.0250, 0.0250 (1) | 27.0,  27.0 (1) | 0.193,  0.193 (1) | 119±120,  119 (2) |
|  | liver | 140±92.2,  87.0 (3) | 0.0875±  0.0884, 0.0875 (2) | 17.2±22.2,  7.86 (4) | 511±569,  210 (3) | 394±324,  233 (3) | 2.91±3.63,  1.35 (4) | 1.65±2.05,  1.65 (2) | 585±1012,  115 (4) | 0.275±  0.177,  0.275 (2) | 114±94.5,  74.0 (4) |
| Adult Male | kidney | 855,  855 (1) | - | 8.86±4.83,  8.92 (3) | 602±288,  733 (3) | 349,  349 (1) | 1.47±  0.151,  1.45 (3) | 0.200±0,  0.200 (3) | 9.42±5.66,  11.0 (3) | - | 110±78.8,  100.4 (3) |
|  | liver | 307±266,  307 (2) | 0.150±0, 0.150 (2) | 35.4±27.4,  27.0 (5) | 708±485,  967 (5) | 275±194,  275 (2) | 2.77±1.66,  2.80 (5) | 1.02±  0.982,  0.867 (4) | 235±176,  207 (5) | 0.562±  0.583,  0.562 (2) | 344±181,  283 (5) |
| Adult Unknown | kidney | - | - | - | - | - | - | - | - | - | - |
|  | liver | - | 0.150, 0.150 (1) | 43.3,  43.3 (1) | 433,  433 (1) | - | 7.00,  7.00 (1) | 0.200,  0.200 (1) | 533,  533 (1) | 1.57,  1.57 (1) | 433,  433 (1) |
| Juvenile | kidney | 247,  247 (1) | 0.138±  0.159,  0.138 (2) | 8.50±2.12,  7.50 (4) | 257±183,  173 (3) | 767,  767 (1) | 2.36±  0.560,  2.23 (3) | 0.0883±  0.0970,  0.0400 (3) | 12.9±11.2,  8.00 (3) | 0.403±  0.325,  0.403 (2) | 104±38.9,  106 (4) |
|  | liver | 142±196,  54.0 (3) | 0.120±  0.0760, 0.150 (6) | 53.1±38.1,  41.8 (7) | 666±444,  583 (7) | 475±269,  533 (3) | 8.29±3.77, 7.60 (7) | 0.559±  0.557,  0.243 (6) | 95.5±205,  19.3 (7) | 0.928±  0.677,  1.02 (5) | 482±350,  418 (7) |
| Calf | kidney | 467,  467 (1) | 0.0250,  0.0250 (1) | 15.5±6.51,  11.0 (7) | 483±129,  500 (7) | 367,  367 (1) | 2.18±1.47,  1.63 (7) | 0.175±  0.0661,  0.200 (7) | 2.48±  0.501,  2.33 (5) | 0.180, 0.180 (1) | 78.1±19.9,  70.0 (7) |
|  | liver | 523±807,  156 (4) | 0.150±0, 0.150 (7) | 117±103,  80.0 (17) | 817±401,  767 (17) | 726±1021,  342 (4) | 7.03±6.18,  5.00 (17) | 0.200±0,  0.200 (14) | 5.37±4.21,  4.32 (14) | 0.286±  0.360,  0.150 (7) | 199±185,  122 (17) |
| **Ecotype** | | | | | | | | | | | |
| Offshore | kidney | - | - | - | - | - | - | - | - | - | - |
|  | liver | 70.0±  24.0,  70.0 (2) | 0.150, 0.150 (1) | 16.0±  12.11,  18.0 (3) | 609±580,  410 (3) | 192±58.7,  192 (2) | 4.57±4.66,  2.50 (3) | 0.200,  0.200 (1) | 21.7±32.7,  3.75 (3) | 0.150,  0.150 (1) | 225±307,  58.0 (3) |
| Resident | kidney | 2400±  2734,  2400 (2) | 0.100±  0.130,  0.0250 (3) | 13.8±8.07,  13.7 (7) | 511±249,  500 (6) | 517±212,  517 (2) | 1.63±  0.296,  1.63 (6) | 0.115±  0.0933,  0.120 (6) | 8.50±8.73,  5.00 (7) | 0.182±  0.0102,  0.180 (3) | 126±60.9,  121 (7) |
|  | liver | 557±788,  247 (4) | 0.116±  0.0703, 0.150 (9) | 92.2±87.0,  70.0 (11) | 892±443,  967 (11) | 1058±781,  738 (4) | 8.97±7.21,  8.31 (11) | 1.03±1.02,  0.609 (10) | 267±629,  19.3 (11) | 0.202±  0.0888,  0.150 (8) | 400±294,  295 (11) |
| Transient | kidney | 670±262,  670 (2) | - | 10.5±6.03,  9.96 (4) | 490±201,  533 (3) | 219±184,  219 (2) | 2.15±2.15,  1.27 (4) | 0.200±0,  0.200 (3) | 6.08±6.98,  2.27 (3) | - | 71.1±31.3,  75.0 (4) |
|  | liver | 164±186,  87.0 (5) | 0.150±0, 0.150 (6) | 82.2±102,  43.3 (15) | 602±427,  595 (14) | 183±148,  182 (5) | 3.90±2.45,  4.33 (15) | 0.200±0,  0.200 (11) | 101±156,  17.1 (12) | 0.936±  0.664,  1.04 (6) | 180±151,  107 (15) |
| Unknown Ecotype | kidney | 247,  247 (1) | 0.0250,  0.0250 (1) | 10.1±1.69,  10.7 (5) | 364±130,  367 (5) | 767,  767 (1) | 2.23±  0.590,  2.23 (5) | 0.165±  0.0783,  0.200 (5) | 14.3±16.1,  14.3 (2) | 0.633,  0.633 (1) | 72.0±14.1,  66.7 (5) |
|  | liver | 367,  367 (1) | 0.150±0, 0.150 (2) | 71.0±34.0,  80.0 (5) | 796±187,  800 (5) | 533,  533 (1) | 7.83±3.92,  7.26 (5) | 0.200±0,  0.200 (5) | 116±249,  5.00 (5) | 1.25±  0.322,  1.25 (2) | 318±270,  172 (5) |
| **Population** |  | | | | | | | | | | |
| Alaska Resident | kidney | - | - | - | - | - | - | - | - | - | - |
|  | liver | - | 0.215, 0.215 (1) | 103,  103 (1) | 1467,  1467 (1) | - | 10.0,  10.0 (1) | 0.285,  0.285 (1) | 33.3,  33.3 (1) | 0.215,  0.215 (1) | 1000,  1000 (1) |
| Southern Resident | kidney | 467,  467 (1) | 0.0250,  0.0250 (1) | 12.6±9.40,  10.5 (4) | 711±102,  733 (3) | 367,  367 (1) | 1.41±  0.195,  1.33 (3) | 0.142±  0.101,  0.200 (3) | 6.30±3.89,  5.57 (4) | 0.180,  0.180 (1) | 116±64.0,  117 (4) |
|  | liver | 867±  1216,  867 (2) | 0.120±  0.0600, 0.150 (4) | 107±119,  54.8 (6) | 901±449,  838 (6) | 1465±  1068,  1465 (2) | 8.34±10.1,  4.58 (6) | 1.00±  0.851,  0.933 (5) | 129±212,  14.4 (6) | 0.150±0,  0.150 (3) | 267±171,  207 (6) |
| Northern Resident | kidney | 4333,  4333 (1) | 0.0250,  0.0250 (1) | 19.2±4.95,  19.2 (2) | 400±0,  400 (2) | 667,  667 (1) | 1.83±  0.283,  1.83 (2) | 0.113±  0.124,  0.113 (2) | 14.7±17.4,  14.7 (2) | 0.193,  0.193 (1) | 137±94.3,  137 (2) |
|  | liver | 247±0, 247 (2) | 0.108±  0.0722, 0.150 (3) | 66.0±16.8,  64.6 (3) | 894±333,  991 (3) | 650±165,  650 (2) | 9.32±1.72,  8.33 (3) | 1.17±1.67,  0.200 (3) | 703±1210,  5.39 (3) | 0.233±  0.144,  0.150 (3) | 310±65.9,  295 (3) |
| Resident  (unspecified) | kidney | - | 0.250,  0.250 (1) | 7.67,  7.67 (1) | 130,  130 (1) | - | 1.87,  1.87 (1) | 0.0400,  0.0400 (1) | 5.00,  5.00 (1) | 0.173,  0.173 (1) | 147,  147 (1) |
|  | liver | - | 0.0250, 0.0250 (1) | 70.0,  70.0 (1) | 260,  260 (1) | - | 10.7,  10.7 (1) | 1.53,  1.53 (1) | 19.7,  19.7 (1) | 0.250,  0.250 (1) | 867,  867 (1) |
| Offshore | kidney | - | - | - | - | - | - | - | - | - | - |
|  | liver | 70.0±  24.0,  70.0 (2) | 0.150, 0.150 (1) | 16.0±12.1,  18.0 (3) | 609±580,  410 (3) | 192±58.7,  192 (2) | 4.57±4.66,  2.50 (3) | 0.200,  0.200 (1) | 21.7±32.7,  3.75 (3) | 0.150,  0.150 (1) | 225±307,  58.0 (3) |
| Gulf of Alaska Transient | kidney | - | - | - | - | - | - | - | - | - | - |
|  | liver | - | 0.150, 0.150 (1) | 157±160,  157 (2) | 650±306,  650 (2) | - | 5.33±2.36,  5.33 (2) | 0.200±0,  0.200 (2) | 533,  533 (1) | 1.57,  1.57 (1) | 270±231,  270 (2) |
| West Coast Transient | kidney | - | - | 14.7±5.19,  14.7 (2) | 600±  94.3,  600 (2) | - | 3.22±2.99,  3.22 (2) | 0.200±0,  0.200 (2) | 2.05±  0.306, 2.05 (2) | - | 75.0±25.9,  75.0 (2) |
|  | liver | 92.5±  37.5,  92.5 (2) | 0.150±0, 0.150 (3) | 86.0±110,  42.6 (8) | 641±491,  689 (8) | 69.9±96.3,  69.9 (2) | 4.18±2.81,  4.33 (8) | 0.200±0,  0.200 (6) | 17.8±26.9,  8.78 (6) | 0.975±  0.770,  1.10 (3) | 159±136,  116 (8) |
| Transient NT1 haplotype | kidney | 855,  855 (1) | - | 8.92,  8.92 (1) | 271,  271 (1) | 349,  349 (1) | 1.45,  1.45 (1) | 0.200,  0.200 (1) | 14.1,  14.1 (1) | - | 100.4,  100.4 (1) |
|  | liver | 495,  495 (1) | - | 6.59,  6.59 (1) | 236,  236 (1) | 412,  412 (1) | 4.40,  4.40 (1) | 0.200,  0.200 (1) | 137,  137 (1) | - | 173,  173 (1) |
| Transient  (unspecified) | kidney | 485,  485 (1) | - | 3.80,  3.80 (1) | - | 89.0,  89.0 (1) | 0.700,  0.700 (1) | - | - | - | 34.0,  34.0 (1) |
|  | liver | 70.5±  23.3,  70.5 (2) | 0.150±0, 0.150 (2) | 56.4±71.7,  29.6 (4) | 587±470,  522 (3) | 182±0,  182 (2) | 2.49±1.83,  1.67 (4) | 0.200±0,  0.200 (2) | 108±107,  94.3 (4) | 0.562±  0.583,  0.562 (2) | 178±198,  93.5 (4) |
| Unknown Population | kidney | 247,  247 (1) | 0.0250,  0.0250 (1) | 10.1±1.69,  10.7 (5) | 364±130,  367 (5) | 767,  767 (1) | 2.23±  0.590,  2.23 (5) | 0.165±  0.0783,  0.200 (5) | 14.3±16.1,  14.3 (2) | 0.633,  0.633 (1) | 72.0±14.1,  66.7 (5) |
|  | liver | 367,  367 (1) | 0.150±0, 0.150 (2) | 71.0±34.0,  80.0 (5) | 796±187,  800 (5) | 533,  533 (1) | 7.83±3.92,  7.26 (5) | 0.200±0,  0.200 (5) | 116±249,  5.00 (5) | 1.25±  0.322,  1.25 (2) | 318±270,  172 (5) |
