# Supplementary figures and images for: Concentrations of essential and non-essential elements in eastern North Pacific killer whales (Orcinus orca)
Source: PLoS One. 2026 Jul 15;21(7):e0353196. doi: 10.1371/journal.pone.0353196 (PMC13372180; doi:10.1371/journal.pone.0353196)

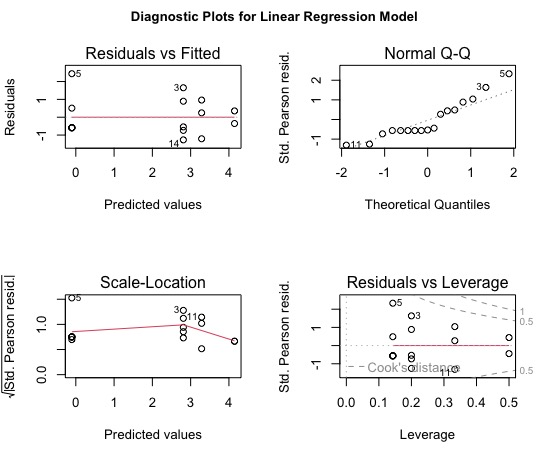

Supplement: S1 Fig — (DOCX) [file pone.0353196.s003.docx]

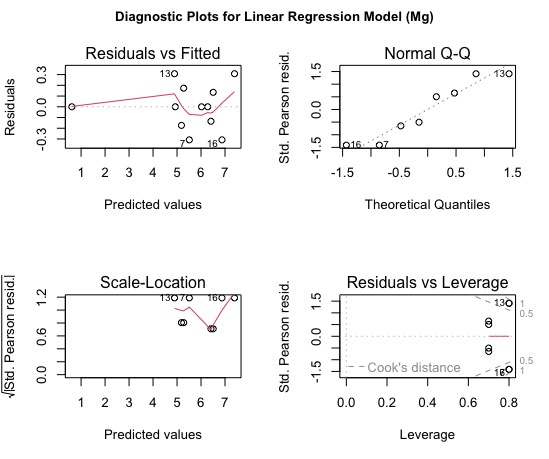


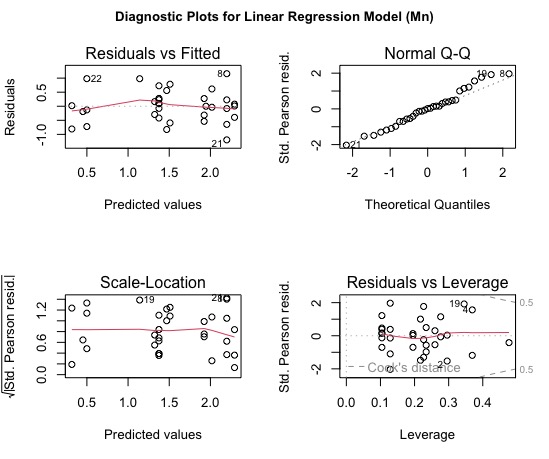

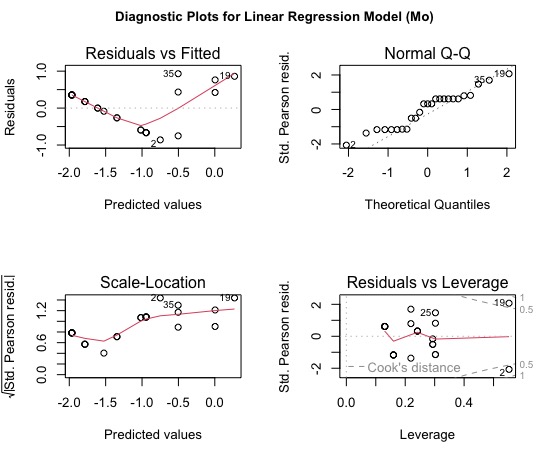

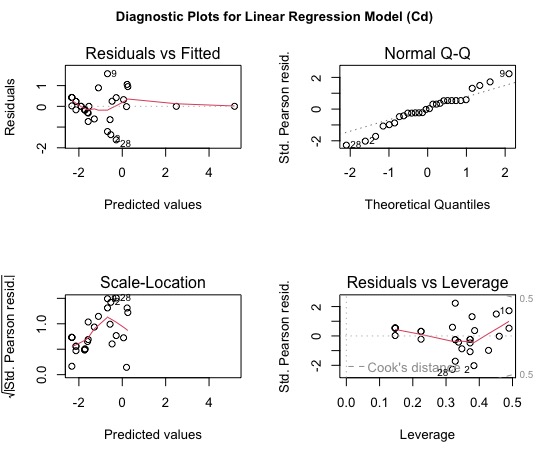

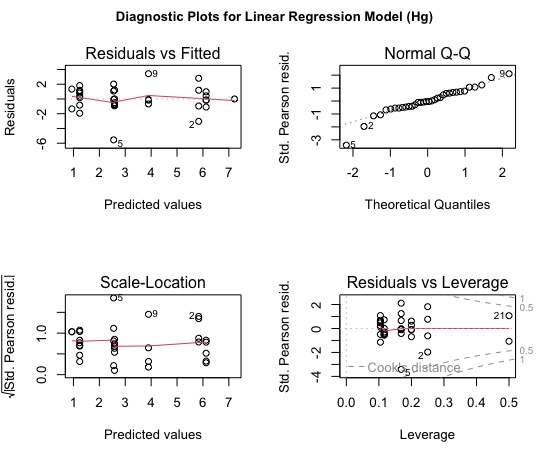

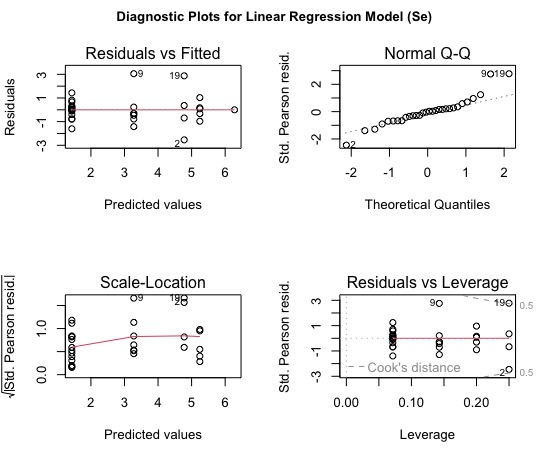

Supplement: S2 Fig — (DOCX) [file pone.0353196.s004.docx]
